
JOURNAL INFORMATION
==============================
Journal ID: Wirtschaftsdienst

Wirtschaftsdienst

EISSN: 1613-978X

ARTICLE INFORMATION
==============================
PMCID: PMC9226282
DOI: 10.1007/s10273-022-3210-8
Article ID: 3210
Article version: 1
Subjects: Zeitgespräch

Hausgemachte Inflationsrisiken

Homemade Inflation Risks

Kooths Stefan stefan.kooths@ifw-kiel.de

grid.462465.7 0000 0004 0493 2817 Leiter Prognosezentrum, Institut für Weltwirtschaft, Kiellinie 66 , 24105 Kiel, Deutschland
Electronic publication date: 2022 Jun 24
Print publication date: 2022
Volume: 102
Issue: 6
First page: 434
Last page: 437
Copyright: © Der/die Autor:in 2022
License: Open Access: Dieser Artikel wird unter der Creative Commons Namensnennung 4.0 International Lizenz veröffentlicht (https://creativecommons.org/licenses/by/4.0/deed.de).
Open Access wird durch die ZBW — Leibniz-Informationszentrum Wirtschaft gefördert.
License URL: https://creativecommons.org/licenses/by/4.0/

Keywords: E31, E58, E65
issue-copyright-statement: © The Author(s) 2022

==============================

Mit der hohen Teuerung ist ein Problem zurück, das die maßgeblichen wirtschaftspolitischen Akteure seit längerem nicht mehr auf der Rechnung hatten. Viele treibt nun die Frage um, ob die Inflation gekommen ist, um zu bleiben. Die Frage so zu stellen, kann in die Irre führen, schwingt dabei doch etwas Schicksalhaftes mit. Inflation ist aber kein Schicksal, sondern das Ergebnis misslungener Stabilitätspolitik. Stabilität bezeichnet ein Wirtschaften im Einklang mit den Möglichkeiten. Werden die Produktionskapazitäten durch zu viel Kaufkraft überstrapaziert, macht sich diese Unwucht preistreibend geltend. Keine neue Erkenntnis, sondern angesichts der wirtschaftspolitischen Reaktionen auf die Corona- und Energiekrise eher eine vergessene, jedenfalls keine veraltete.

## Nachwehen der Coronainterventionen

Der gegenwärtige Inflationsschub bildet im Wesentlichen die Nachwehen zu den massiven fiskal- und geldpolitischen Interventionen, die zur Abfederung der pandemiebedingten Krise nahezu weltweit erfolgten (vgl. Abbildung 1).[1] Die Coronapandemie wirkte über die zum Infektionsschutz ergriffenen Schutzmaßnahmen (Lockdowns und andere Kontaktbeschränkungen) zunächst als interruptiver Schock auf die Weltwirtschaft. Hierdurch brach die ökonomische Aktivität vorübergehend so stark ein wie noch nie seit dem Zweiten Weltkrieg. Die Diagnose eines Schocks, der die wirtschaftliche Aktivität vorübergehend unterbricht, nicht aber die Produktionsstrukturen infrage stellt, verweist unmittelbar auf die Stoßrichtung der angemessenen wirtschaftspolitischen Therapie. Weil die allermeisten Geschäftsmodelle, die vor der Pandemie marktfähig waren, dies auch nach der Pandemie sein dürften, kam es gesamtwirtschaftlich vor allem darauf an, die betroffenen Unternehmen über die Zeit des Stillstands zu bringen, um so wertvolles organisatorisches Wissen zu schützen. Das sprach für gezielte Subventionen an Unternehmen und für Hilfen für ihre Beschäftigten, nicht aber für darüber hinausgehende allgemeine nachfragestützende Maßnahmen (wie hierzulande etwa die temporäre Absenkung des Regelsatzes der Mehrwertsteuer). Denn die Ursache der Krise war kein Nachfragemangel, sondern eine aus gesundheitspolitischen Gründen erzwungene wirtschaftliche Auszeit. Durch die defizitfinanzierten staatlichen Krisenpakete wurde in großem Stil Kaufkraft im privaten Sektor geschaffen, dem keine Güterproduktion gegenüberstand. Diese Phantomeinkommen müssen ihrer Natur nach inflationär wirken, sobald die Akteure zu ihren gewohnten Aktivitätsmustern zurückkehren können.

Die im Zuge von Produktionsausfällen entgangenen Einkommen kann der Staat gesamtwirtschaftlich nicht ausgleichen. Er kann nur die Lasten anders verteilen. Das Ausweichen in Haushaltsdefizite verschleiert diesen Umstand. So haben die Masseneinkommen in Deutschland im Krisenjahr 2020, in dem die Wirtschaftsleistung um 4,9 % einbrach, real mit 2,4 % zugenommen und auch für die verfügbaren Einkommen aller privaten Haushalte blieb noch ein leichtes Plus. Weil viele Konsummöglichkeiten während der Pandemie verschlossen waren, staute sich Kaufkraft von rund 200 Mrd. Euro in Form von Extra-Ersparnissen auf, was mehr als 10 % der jährlichen Konsumausgaben entspricht.

Fiskalisch ähnelt die durch die Coronapandemie entstandene Lage am ehesten der Wiedervereinigungsphase. Anfang der 1990er Jahre wurde – zunächst durch die Währungsumstellung, später durch staatliche Transfers von West nach Ost – in großem Umfang Kaufkraft geschaffen, der keine Güterproduktion gegenüberstand. Allerdings gab es damals gegenüber heute zwei gravierende Unterschiede: Zum einen war der Wiedervereinigungseffekt auf Deutschland beschränkt, sodass über den Außenhandel ein erheblicher Teil des Kaufkraftüberhangs abgefangen werden konnte. Zum anderen hatte die Bundesbank stabilisierend gegengesteuert.

Die Geldpolitik hat demgegenüber während der Coronakrise die Zeichen der Zeit nicht erkannt oder nicht danach gehandelt. Als wäre Nachfragemangel und nicht

1 Vgl. COVID-19 Government Response Tracker, Oxford University, https://www.bsg.ox.ac.uk/research/research-projects/covid-19-government-response-tracker; Policy Responses to COVID-19, International Monetary Fund, https://www.imf.org/en/Topics/imf-and-covid19/Policy-Responses-to-COVID-19 (30. Mai 2022).

Abbildung 1

**Weltweite Coronahilfen für Beschäftigte am Ende des ersten Pandemiejahres**

Die Einkommensunterstützung wird erfasst, wenn die Regierung die Gehälter von Menschen, die ihren Arbeitsplatz verlieren oder nicht arbeiten können, übernimmt oder direkte Barzahlungen, ein universelles Grundeinkommen oder Ähnliches leistet.

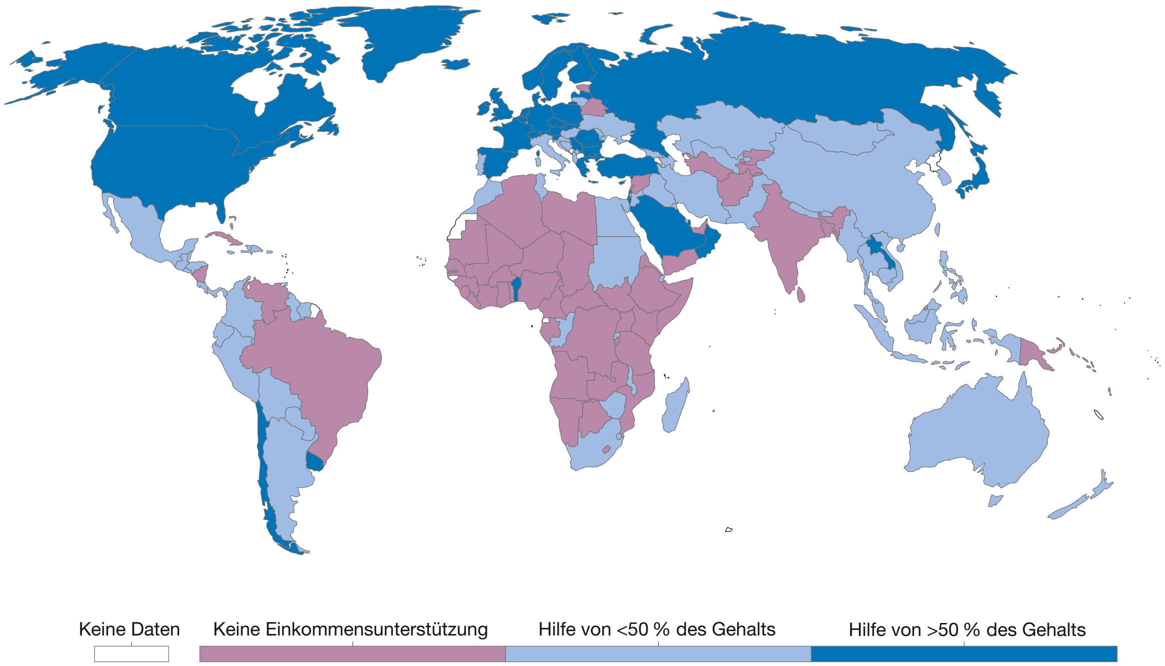

Diese Einkommensunterstützung gilt möglicherweise nicht für Beschäftigte in allen Sektoren und kann auf regionaler Ebene variieren.

Quellen: Oxford COVID-19 Government Response Tracker, Blavatnik School of Government, University of Oxford, ourworldindata.org.

die Produktionsunterbrechung das Kennzeichen der Coronakrise, haben die Notenbanken die Geldschleusen weit geöffnet und im Euroraum (wie auch anderswo) staatliche Defizite, die – abgesehen von unnötig stimulierenden Maßnahmen – stabilitätspolitisch vertretbar waren, über die Notenpresse finanziert. Auf diese Weise konnten auch die übermäßigen Ersparnisse nicht über den Kapitalmarkt gebunden werden, sondern blieben als täglich verfügbare Mittel erhalten (vgl. Abbildung 2). Weil der Staat als Kreditnehmer das neugeschöpfte Zentralbankgeld bei monetärer Staatsfinanzierung unmittelbar in den Wirtschaftskreislauf einspeist, bedarf es für die Geldmengenausdehnung keiner Beteiligung der Geschäftsbanken als Kreditschöpfer. Ihre Rolle bestand lediglich darin, neu emittierte staatliche Wertpapiere zu erwerben und sie an die Notenbanken weiterzureichen. So wurde die Formalität gewahrt, dass die Notenbanken nur auf dem Sekundärmarkt intervenieren, was allerdings für die monetären Effekte ohne Belang ist. Im Ergebnis zog die Euro-Geldmenge M3 sprunghaft an – nach zwölf Monaten lag sie bereits 12,5 % über dem Vorkrisenniveau (Anstieg um 1,5 Billionen Euro von Februar 2021 gegenüber Februar 2020). Weil der Kapitalmarkt dabei netto nicht in Anspruch genommen wurde, blieben die Finanzierungskonditionen für alle Mitgliedsländer des Euroraums weiterhin äußerst günstig.

## Inflation nicht nur von Energiekosten getrieben

Über gestresste Lieferketten wirkt die Coronakrise weiterhin nach. Mit dem russischen Überfall auf die Ukraine bekam der Preisauftrieb vor allem über die Rohstoffmärkte weitere Nahrung. Das akzentuiert den Inflationsprozess, der aber lange vor dem Kriegsausbruch in Gang gekommen war und auch kein reines Energiethema ist. Das gilt in besonderem Maße für die USA, aber auch hierzulande wird die hausgemachte Teuerung immer spürbarer. So zog der Deflator der Bruttowertschöpfung im vergangenen Jahr mit 2,8 % so kräftig an wie seit fast 30 Jahren nicht mehr. Dabei dürfte auch eine Rolle spielen, dass die Produktion nicht nur durch Materialengpässe behindert wird, sondern in zunehmendem Maße durch Personalmangel, der sich mittlerweile bei insgesamt noch unterausgelasteten Kapazitäten quer durch alle Wirtschaftsbereiche zeigt. Ausweislich von Unternehmensbefragungen hat der Fachkräftemangel in Deutschland bereits im vergangenen Jahr historische Höchststände erreicht (Sauer und Wollmershäuser,

Abbildung 2
**Geldmenge M3 im Euroraum**

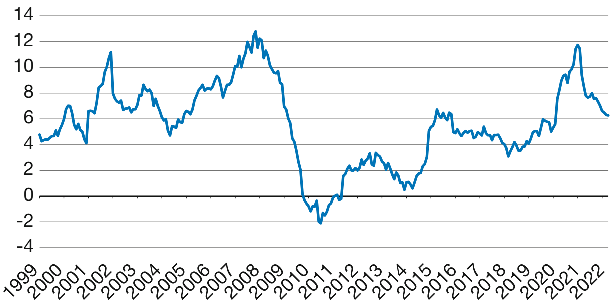

Monatsdaten, saisonbereinigt. Veränderung gegenüber Vorjahr in % (jeweils Stand am Monatsende).

Quelle: Europäische Zentralbank, eigene Berechnungen.

2021), obwohl die Wirtschaftsleistung noch deutlich unter dem Vorkrisenniveau lag.

Die akut hohen Inflationsraten spiegeln gleichwohl in erheblichem Maße die Verteuerung von Energierohstoffen wider. Hierin kommt eine physische Verknappung bzw. unsicherer gewordene Versorgung zum Ausdruck. Bezeichnend ist die wirtschaftspolitische Reaktion. Statt die Preissignale wirken zu lassen, um angemessen mit der Knappheitssituation umgehen zu können, werden ringsum breit angelegte „Entlastungspakete“ auf den Weg gebracht. Während Hilfen für Bedürftige zum Schutz des Existenzminimums notwendig erscheinen, ist eine Entlastung der Bevölkerung in der Breite durch den Staat nicht nur nicht möglich, weil er sich aus der Breite der Bevölkerung finanziert, sondern sogar kontraproduktiv. Denn gesamtwirtschaftlich injiziert der Staat über neue Defizite neue Kaufkraft in den Privatsektor, die die Inflation weiter anfacht. Den Einkommensschwachen erweist er damit einen Bärendienst, weil sich auch deren Lebenshaltung so weiter verteuert. Maßnahmen, die insbesondere die Energienachfrage stützen (etwa durch Energiesteuersenkungen oder Energiekostensubventionen), treiben nicht zuletzt die Nettopreise für Energie. Das entlastet die Energieverbrauchenden nicht, sondern füttert die Energielieferanten. Abermals passt die wirtschaftspolitische Reaktion nicht zur Diagnose des gesamtwirtschaftlichen Problems.

Dies gilt auch für das Agieren auf EU-Ebene, sowohl finanz- wie geldpolitisch. Fiskalisch war der Coronaschock kein Anlass für zentralisiertes Handeln, denn es bestand kein nationaler Interessengegensatz im Sinne einer Trittbrettfahrerproblematik oder starker grenzüberschreitender Spillover-Effekte (Felbermayr und Kooths, 2020). Jedes Land hatte ausreichende Anreize, die marktfähigen Unternehmen über die pandemiebedingte Durststrecke zu bringen. Zur länderübergreifenden Koordination hätte die EU-Kommission darauf achten müssen, dass dysfunktionale Subventionen unterbleiben, die im Binnenmarkt wettbewerbsverzerrend wirken. Da während der Pandemiephase in allen Ländern in erheblichem Umfang Kaufkraft aufgestaut wurde, war auch früh klar, dass der Erholungsprozess selbsttragend sein würde. Das „Next-Generation-EU“-Fiskalprogramm mit seiner Aufbau- und Resilienzfazilität steht daher stabilisierungspolitisch auf schwachen Beinen. Es wird vielmehr stimulierend wirken, wenn sich die Krise von selbst zurückbildet, und dann vor allem diejenigen Bereiche anregen, die von der Krise gar nicht betroffen waren, wie etwa die Digitalbranche oder die Bauwirtschaft. Bei angespannten Kapazitäten wird dieser Stimulus tendenziell inflationär zum Tragen kommen.

## Europäische Zentralbank noch immer zu passiv

Ebenso wenig passt das Stillhalten der Geldpolitik im Euroraum in die gesamtwirtschaftliche Landschaft. Nichtagieren bedeutet bei anziehender Inflation, den Expansionsgrad der Geldpolitik zu erhöhen, weil es die Realzinsen noch weiter in den negativen Bereich drückt. Konjunkturell ist geldpolitischer Attentismus nicht zu begründen, wenn die Produktion die Nachfrage nicht bedienen kann. Zudem schwimmen die Geschäftsbanken nach Jahren ultraexpansiver Geldpolitik in historisch einmaliger Überschussliquidität. Von dieser Seite besteht eine immense Geldschöpfungskapazität. Kein Wunder, dass sich bereits Stimmen aus Bankkreisen mit der Forderung melden, die regulatorischen Kapitalpuffer abzuschmelzen, um eine massive Kreditexpansion in Gang zu setzen. Solche Forderungen korrespondieren mit einer Notenbankkommunikation, die ihren geldpolitischen Kurs mit der Sorge begründet, die Konjunktur nicht abwürgen zu wollen. Neben einer Normalisierung der Leitzinsen ist daher eine Absorption der hohen Zentralbankliquidität vordinglich.

Fiskalische Abstinenz auf EU-Ebene und energisches Gegensteuern durch die EZB setzen Mitgliedsländer voraus, die unbeschränkt kapitalmarktfähig sind. Das meint einen Zustand der Staatsfinanzen, deren Schuldentragfähigkeit auch in einer schweren Wirtschaftskrise nicht infrage steht, sodass die Länder ihre Refinanzierung ohne gravierende Risikoaufschläge über die Emission von Wertpapieren ohne Eingriffe der Notenbank bewältigen können. Daran bestehen mittlerweile bei einigen großen Mitgliedsländern Zweifel, wodurch das sonst kaum zu plausibilisierende Handeln auf Gemeinschaftsebene zu erklären wäre. Für die weitere Inflationsentwicklung ist dies vor allem mit Blick auf die permissive Ausrichtung der Geldpolitik ein Grund für ernste Besorgnis, weil so die hohe Staatsverschuldung die Handlungsfähigkeit der Geldpolitik einschränkt, die aus Sorge vor steigenden Risikoprämien (und damit drohenden Schuldenkrisen) unter die fiskalische Dominanz gerät.

Demografie und Dekarbonisierung lassen kapitalmarktseitig in den kommenden Jahren tendenziell steigende Zinsen erwarten. Denn zunehmend älter werdende Gesellschaften drosseln ihre Ersparnis, während der erfolgreiche Übergang zu emissionsneutraler Wirtschaftsweise weltweit massive Investitionen erfordert, die auf absehbare Zeit die Produktionskapazitäten nicht erweitern, da der Kapitalstock eher umgebaut als vergrößert wird (Projektgruppe Gemeinschaftsdiagnose, 2022). Die spezielle Konstellation der Babyboomer verschärft zudem die Verteilungskonflikte um Gegenwartsgüter, was zusätzlich zinssteigernd wirkt. Wenn die Geldpolitik diesen realwirtschaftlich angelegten Zinstrend nicht nachvollzieht, sondern dem aus Rücksicht auf fragile Staatsfinanzen entgegenwirkt, kann sich die dann eintretende Überforderung der Produktionsmöglichkeiten nur über die Preisventile entladen.

### Geld- und Fiskalpolitik am besten Hand in Hand

Der Inflationsschub nach Corona wird mit dem Abbau des Kaufkraftüberhangs wieder abflauen, vorausgesetzt, die Wirtschaftspolitik schafft keine neuen Inflationstreiber. Entscheidend sind daher jetzt geldpolitische Signale, die einem Szenario entgegenwirken, in dem sich die Inflationserwartungen nicht zuletzt mit Blick auf die drohende fiskalische Dominanz entankern. Käme es dazu, verselbständigt sich der heimische Preisauftrieb. Denn dann werden auch die Tarifpartner höhere Lohnabschlüsse vereinbaren, weil die Beschäftigten ihre Reallöhne absichern wollen und die Unternehmen davon ausgehen können, bei der Überwälzung nicht eingebremst zu werden. Was dann aussieht wie eine Lohn-Preis-Spirale, ist tatsächlich eine Preiserwartungs-Lohn-Spirale. Ein Wiedererlangen der geldpolitischen Glaubwürdigkeit würde später ein umso größeres Gegensteuern erfordern. Eine solche Neuauflage der Volcker-Lektion sollte man sich ersparen.

Inflation ist und bleibt ab der mittleren Frist ein monetäres Phänomen. Das nimmt die Fiskalpolitik aber nicht aus der Verantwortung. Die Finanzpolitik kann es der Notenbank schwerer oder leichter machen, ihrem Auftrag zur Preisstabilität nachzukommen. Sie hat es in der Hand, durch stabilitätsgerechtes Handeln der Geldpolitik den materiellen Freiraum zu schaffen, den sie braucht. Dazu gehört kurzfristig eine stärkere Ausrichtung an den gesamtwirtschaftlichen Möglichkeiten sowie perspektivisch eine Bereinigung der Überschuldungsproblematik im Euroraum. Hierfür sind dicke Bretter zu bohren, auch wenn Vorschläge dazu schon seit Jahren auf dem Tisch liegen (Snower et al., 2013). Das setzt freilich den Konsens voraus, den Euroraum als Hartwährungssystem erhalten zu wollen.

The COVID-19 pandemic massively interrupted economic activity all over the world. Governments responded by running huge fiscal deficits (financed via central banks) to support firms and consumers, thereby injecting purchasing power into the private sector on a large scale. With no corresponding production of goods and services, these phantom incomes are by their very nature inflationary. This explains the root cause of the post-pandemic price pressure. New fiscal programs created to compensate resulting losses of purchasing power only prolong the inflationary phase. Anchored inflation expectations are key to keeping the inflation process temporary. While this clearly falls under the responsibility of the monetary authorities, fiscal policymakers should support central banks by quickly ending the crisis mode. This is particularly important for the euro area where the problem of fiscal dominance would otherwise continue to increase.

Prof. Dr. Stefan Kooths ist Direktor des Forschungszentrums Konjunktur und Wachstum am Kiel Institut für Weltwirtschaft (IfW).


Literatur

Felbermayr, G. und S. Kooths (2020), Stabilisierungspolitik in der Corona-Krise, Kiel Policy Brief, 138.
Projektgruppe Gemeinschaftsdiagnose (2022), Von der Pandemie zur Energiekrise — Wirtschaft und Politik im Dauerstress, Frühjahrsgutachten 2022.
Sauer S Wollmershäuser T Fachkräftemangel wird zunehmend zur Belastung für die deutsche Wirtschaft ifo Schnelldienst digital 2021 17 1
Snower, D., J. Boysen-Hogrefe, K.-J. Gern, H. Klodt, S. Kooths, C.-F. Laaser, B. van Roye, J. Scheide und K. Schrader (2013), Kieler Krisen-Kompass: Ein Gesamtpaket zur Überwindung der Krise im Euroraum, Kiel Policy Brief, 58.
